# School and Student Characteristics Associated With Screen-Time Sedentary Behavior Among Students in Grades 5-8, Ontario, Canada, 2007-2008

**Published:** 2010-10-15

**Authors:** Scott T. Leatherdale, Guy Faulkner, Kelly Arbour-Nicitopoulos

**Affiliations:** Department of Population Studies and Surveillance, Cancer Care Ontario. Dr Leatherdale is also affiliated with the Department of Health Studies and Gerontology, University of Waterloo, Waterloo, Ontario, Canada, and the Dalla Lana School of Public Health, University of Toronto, Toronto, Ontario, Canada.; Department of Health Studies and Gerontology, University of Waterloo, Waterloo, Ontario, Canada; Department of Health Studies and Gerontology, University of Waterloo, Waterloo, Ontario, Canada

## Abstract

**Introduction:**

We examined school and student characteristics associated with screen-time sedentary behavior.

**Methods:**

We analyzed data collected from 2,449 students in grades 5 through 8 who attended 30 elementary schools in Ontario, Canada. We used multilevel logistic regression to examine the student- and school-level factors associated with moderate and high screen-time sedentary behavior.

**Results:**

Moderate screen time did not vary significantly across schools. Student characteristics significantly associated with moderate screen time were sex, number of friends who are active, and parental encouragement of physical activity. High screen time did vary significantly across schools; school-level differences accounted for 12% of the variability in the odds of a student reporting high screen time. Students who attended a school in the more advanced phase of emphasizing participation in physical activity through school programs were less likely to report high screen time compared with students who attended schools in the earlier phase for this school-level indicator. Student characteristics significantly associated with high screen time were sex, parental encouragement of physical activity, parental support of physical activity, and race/ethnicity.

**Conclusions:**

High levels of screen-time sedentary behavior are associated with both student characteristics and the characteristics of the school a student attends. Developing a better understanding of the school characteristics associated with sedentary behavior will be valuable for guiding the development of interventions to reduce sedentary behavior among youth populations.

## Introduction

The negative effect of sedentary lifestyles on children's health is a source of concern (1); the increasing prevalence of obesity among North American youths coincides with an increasing prevalence of high screen time (typically defined as any combination of activities such as watching television or playing video games) in this age group (2). National organizations have developed recommendations to limit sedentary behavior among youths (3). For instance, the American Academy of Pediatrics recommends that children's screen time be limited to no more than 1 to 2 hours of quality programming per day (3). Few children or adolescents meet these guidelines (4), and activities designed to reduce sedentary behavior in this age group should be a public health priority.

Defining sedentary behavior in terms of the absence of physical activity (PA) (5) fails to acknowledge the range and complexity of sedentary pursuits that can be modified. For example, research has focused on the relationship of obesity and PA to television watching (6) and more recently to other types of screen-time sedentary behavior, such as playing video games and using a computer (7). Research suggests that most children spend 1 to 3 hours in these types of screen-time sedentary behaviors per day (1).

Social cognitive theory (8) posits that sedentary behavior is influenced by personal beliefs (believing parents do not encourage PA), physical characteristics (being overweight), and other related behaviors (frequency of regular participation in PA). Empirical research has demonstrated support for these relationships with respect to screen time (9,10).

An ecologic approach to youth inactivity acknowledges that sedentary behaviors are a function of not only individual characteristics but also context (11). The school environment may be associated with the time youths spend in screen-time sedentary behavior (11). We still know very little about the associations between the school environment and sedentary behavior, except that 1) youths spend approximately 25 hours per week in school throughout the school year, 2) school-based interventions to reduce sedentary behavior are likely more effective than individual-based interventions (12), 3) the most prevalent behavior among youths after school is screen-time sedentary behavior (13), and 4) other behaviors, such as smoking (14) and PA (15) vary among elementary schools. We sought to better understand through multilevel analyses the school- and student-level characteristics associated with screen-time sedentary behavior among children in grades 5 through 8. Such insight would be valuable to appropriately tailor school-based interventions to reduce sedentary behavior.

## Methods

### Design

This cross-sectional study used data collected from November 2007 through April 2008 as part of the PLAY-Ontario (PLAY-On) study. PLAY-On used a convenience sample of students in grades 5 through 8 who attended 30 elementary schools in Ontario, Canada. Schools were purposefully recruited to make up a sample that covered the major geographic regions of Ontario and urban and rural areas; budget constraints precluded random sampling of schools. Student-level data were collected from consenting students by using a previously validated student PA questionnaire (16). Additional details about PLAY-On are available online (www.shapes.uwaterloo.ca/projects/playon).

School-level data were collected by using the PA categories of the elementary school version of the School Health Environment Survey (SHES) (17). The SHES tool used in PLAY-On is designed to assess programs, activities, committees, facilities, and guidelines related to PA in the school environment. The 4 PA categories in the SHES tool are aligned with the government of Ontario's Foundations for a Healthy School (FHS) (18). Additional details about the SHES measures and assessment categories are available in print (17) and online (www.healthyschoolplanner.uwaterloo.ca).

### Data collection

All students at the participating schools were eligible to participate if they obtained active consent from their parents, and students could decline to participate at any time. Eligible students completed the student PA questionnaire during class time. At each participating school, the administrator(s) most knowledgeable about the school's programs, policies, and resources was asked to complete the SHES tool. The University of Waterloo Office of Research Ethics and appropriate school board ethics committees approved the study procedures.

### Participants

Of the 4,838 students enrolled in grades 5 through 8 at the 30 participating elementary schools, 2,449 (51%) completed the survey; missing responses resulted from parent or student refusal (n = 2,082) and absenteeism on the day of the survey (n = 307). This response rate is consistent with other active-consent studies with Canadian elementary school students (19). All 30 elementary schools completed the SHES survey.

### Measures

Respondents were asked to report the number of hours for each day of the week that they spent watching television or movies or playing video or computer games. We calculated the average screen time per day based on the average time reported during the previous week for each construct. Consistent with existing research and national guidelines (3,5,20), we then grouped responses into 3 categories: less than 1 hour per day (low screen time), 1 to 3 hours per day (moderate screen time), and more than 3 hours per day (high screen time).

Using previously validated self-report measures (16), body mass index (BMI) was calculated from weight in kilograms divided by height in meters squared. Consistent with guidelines and growth charts from the Centers for Disease Control and Prevention (CDC) (21), students in the 6th through 84th percentile for BMI adjusted for age and sex were classified as normal weight, students in the 85th through 94th percentile as overweight, and students in the highest 5th percentile as obese. Students in the lowest 5th percentile were excluded from analysis. For the multivariate analyses, students classified as overweight (n = 186) or obese (n = 110) were collapsed into a single category (overweight) to represent all youths who may be at risk for diseases associated with being overweight. We used previously validated self-report measures (16) to measure PA by asking respondents how many minutes of vigorous PA (VPA) (ie, "physical activities that increase your heart rate and make you breathe hard and sweat such as jogging or team sports") and moderate PA (MPA) (ie, lower-intensity physical activities such as walking or biking to school) they engaged in on each of the previous 7 days. The average kilocalories per kilogram of body weight per day (KKD) expended in VPA and MPA were calculated as follows:

Equation

KKD = [(Hours of VPA * 6MET) + (Hours of MPA * 3MET)] / 7 days

This equation assumes that the standard metabolic equivalent (MET, a unit used to estimate the amount of oxygen used by the body during PA) for VPA is 6 and for MPA is 3 in accordance with CDC guidelines (www.cdc.gov/nccdphp/dnpa/physical/terms).

Because youths tend to substantially overreport time spent doing PA in self-report (16,22), it is more meaningful to compare the relative PA levels of students in the sample rather than using predetermined cutpoints (eg, <3 KKD, 6-8 KKD) to classify students' PA levels (16). Therefore, consistent with previous research (5,9), students who were 1 standard deviation below the sample mean for KKD (≤16th percentile) were classified as low active, students 1 standard deviation above the sample mean for KKD (≥84th percentile) were classified as high active, and students within 1 standard deviation above or below the sample mean for KKD (17th to 83rd percentile) were classified as moderately active.

The measures for social influences were consistent with previous research (9,20). Respondents reported how much their parent(s) or guardian(s) encourage them to be physically active (strongly encourage, encourage, do not encourage), how much their parent(s) or guardian(s) support them in being physically active (eg, driving them to team games, buying them equipment) (very supportive, supportive, unsupportive), and how many of their 5 closest friends are physically active (0-5). Race/ethnicity was classified by asking students to report how they would describe themselves (aboriginal, white, Chinese, South Asian, black, Filipino, Latin American, Southeast Asian, Arab, West Asian, Korean, Japanese, or other). Based on the response distribution, respondents were categorized as white or nonwhite.

Consistent with the Ontario Ministry of Education’s 4 FHS components (18), the SHES physical activity tool measured indicators associated with 1) healthy physical environment (availability of, access to, and adequacy in meeting student needs for indoor and outdoor facilities; equipment and resources for safe, quality PA on or near school grounds, both during and outside of school hours), 2) instruction and programs (availability, delivery, and characteristics of curricular physical education; extracurricular PA programs; and active transportation to school, including barriers to implementing such programs), 3) supportive social environment (characteristics of the school’s social environment that predispose, reinforce, and enable enjoyable, lifelong participation in PA or that hinder such activities), and 4) community partnerships (accessibility and availability of support services for PA, which may include partnerships with public health units and community-based services and resources). Each indicator was assigned a classification based on the corresponding phase of implementation: initiation (falls short or exhibits extensive room for improvement in meeting the recommendations related to school capacity for PA), action (meets the recommendations in several but not all areas related to school capacity for PA, exhibits some room for improvement), or maintenance (consistently meets or exceeds the recommendations related to school capacity for PA, encouraged to maintain the current level of commitment to supporting PA at school). Each of the 4 FHS components was also assigned an “overall” phase classification based on the combined responses to component indicators. The assessment schemes for the SHES measures were developed on the basis of recommendations from current research literature, government of Ontario guidelines, and input from experts on PA in schools (17).

### Analyses

Using student-level data, we calculated the prevalence of screen-time levels, PA levels, weight status, race/ethnicity, and social influences, by sex. Using school-level data, we calculated the prevalence of the FHS indicators by the phase of implementation. We then performed 2 multilevel logistic regression analyses; respondents with missing data (n = 158) were excluded from these models. Consistent with previous research (5,9), model 1 differentiated low screen time from moderate screen time, and model 2 differentiated low screen time from high screen time. Significance was set at *P* < .05. The analysis for each model used a 3-step modeling procedure. Step 1 examined whether the differences in the outcome were random or fixed across schools. If significant between-school variation was identified in Step 1, then the analyses proceeded to Step 2, in which a series of univariate analyses examined whether each of the school-level FHS indicators was associated with the outcome; only significant school-level variables identified in Step 2 were retained for further analyses in Step 3. If no significant between-school variation was identified in Step 1, the analyses proceeded directly to Step 3. In Step 3, multivariate models were developed to examine how the student characteristics and the significant school characteristics identified in Step 2 were associated with the outcome. After the final models were developed, we explored contextual interactions between all of the significant school and student characteristics. Statistical analyses were conducted with MLwiN version 2.02 (Centre for Multilevel Modelling, University of Bristol, Bristol, UK).

## Results

The study sample was approximately evenly divided between boys and girls (Table 1), and average age was approximately 12 years. Most students reported 1 to 3 hours of screen time per day; boys were more likely than girls to report 3 or more hours. More boys than girls were highly active. BMI could not be calculated for 47% (n = 1,149) of the sample because of missing height or weight measurements. Among respondents who provided sufficient data to calculate BMI, the mean BMI for boys was 19.5 kg/m^2 ^(standard deviation [SD], 3.8 kg/m^2^) and for girls was 19.1 kg/m^2^ (SD, 4.1 kg/m^2^). Boys were more likely than girls to be overweight or obese, although girls were more likely to have missing BMI data. Most students were white, and significantly more boys than girls were white.

Most schools were in the action phase for healthy physical environment and supportive social environment and the maintenance phase for community partnerships (Table 2). Conversely, most schools were in the initiation phase for instruction and programs. None of the schools was in the maintenance phase for the overall scores for healthy physical environment, instruction and programs, or supportive social environment.

We identified no significant between-school random variation for moderate screen time compared with low screen time. Because the school a student attended was not related to his or her likelihood of reporting moderate screen time, school characteristics were not examined in this model. The student-level characteristics associated with moderate screen time included sex, number of friends who are active, and parental encouragement of PA (Table 3).

We identified significant between-school random variation for high screen time compared with low screen time. Our analysis suggests that school-level differences accounted for 12% of the variability in the odds of a student reporting high screen time. Univariate analyses revealed that 2 school characteristics were associated with the odds of reporting high screen time; however, in the multivariate analyses, only 1 school characteristic remained significant. If a student attended a school that was in the action phase for the indicator "Emphasis placed on maximizing participation in PA through school programs," he or she was less likely to report high screen time than a similar student attending a school that was in the initiation phase for this indicator (Table 3). The student-level characteristics associated with high screen time included sex, parental encouragement of PA, parental support of PA, and race/ethnicity. No significant contextual interactions between school- and student-level characteristics were found.

## Discussion

The students in our sample were highly involved in screen-time sedentary behavior, and our findings provide further support for the recommendation that intervention efforts to reduce sedentary behavior must begin before adolescence (23). Two student-level characteristics were consistently related to screen time. First, boys were more likely than girls to report high screen time. Similar patterns have been found elsewhere (1,11). Second, consistent with research among secondary school students (9,20), we found that parental support and encouragement for PA are associated with screen time among elementary school students. Behavioral theories consistently highlight the important role that influential social models surrounding youths (eg, parents) can have on their behavior (8). In general, social models can influence behavior through modeling, social norms, or providing support and encouragement for the behavior (8). Both direct support (overt provision of assistance) or indirect support (encouragement and emotional support) may be associated with more PA among youths (24); a lack of television rules set by parents may also facilitate more screen time (25). Considering that youth become socialized to be active or inactive by the encouragement and support provided by their parents during the elementary school years (26,27), interventions designed to reduce screen time sedentary behavior should target elementary school children and be tailored to include the participation and involvement of parents whenever possible.

Notably, we found that even when controlling for individual student characteristics, the characteristics of the school a student attends were associated with high levels of screen time. Because youths spend up to 12 years of their life in school, the school environment is an ideal place to promote more active lifestyles in this population. To the best of our knowledge, this is the first study to find that the likelihood of a student reporting levels of screen time that exceed existing public health recommendations (3) was associated with the characteristics of the school he or she attended. Specifically, students were less likely to have high screen time if they attended a school that emphasized participating in PA through school programs. These findings are consistent with existing research (11) and guidelines that recommend schools provide opportunities for students to be active during and after school hours to reduce time spent in sedentary behaviors (12); these opportunities can consist of providing students with access to resources before, during, or after school or working with community partners to ensure students have access to resources and facilities.

School-based strategies designed to alter the school environment have a more pronounced effect on increasing PA levels than do individual approaches targeting knowledge and beliefs (28). For instance, schools can compensate for the increase in sedentary behavior as children get older by offering students the opportunity to participate in other activities such as varsity and intramural teams at school (20). Such activities may be particularly important after school because this is the time of day when sedentary behaviors commonly occur (13). Rewarding student behavior by providing additional supervised areas for children to play during the school day (29) or providing additional after-school programs (30) may promote active rather than sedentary choices during students' discretionary time. Additional research is required to evaluate the effect of such initiatives.

Our findings, together with previously published empirical research (14,31), suggest that program planners should target additional prevention resources to the schools that are putting students at the highest risk for being sedentary (ie, schools that do not encourage activity through school programs). By targeting these "high-risk" schools, intensive prevention programs could be implemented where they are most likely to influence students' behavior (31). It may also be beneficial to tailor programs to the needs of high-risk students. For instance, our finding that nonwhite race/ethnicity is associated with more sedentary behavior suggests that prevention programs may need to be tailored to the needs of nonwhite students.

Limitations to our study should be acknowledged. First, the SHES is designed to assess PA environments and policies. Accordingly, there is a lack of correspondence between the SHES and our outcome of interest, screen-time sedentary behavior. This may explain why school-level differences accounted for modest variability in the odds of a student reporting high screen time. Some school-level factors may have been related to sedentary behavior but not assessed by the SHES.

Nearly half of participants in the host study did not report height or weight, preventing us from calculating their BMI. Previous research on older adolescent populations has also reported large amounts of missing self-reported BMI data and that BMI data are more likely to be missing among younger respondents (32,33). Consistent with those studies, additional analyses performed with the PLAY-On data identified an age-related trend in which the prevalence of missing BMI data was higher for younger respondents (34). Therefore, it is possible that some of the results identified in our study may be biased (eg, the lack of associations between BMI and screen time); the results presented in relation to BMI should be interpreted with caution. The missing BMI values may be a result of motivated nonresponding, which would have important implications for the feasibility of using self-reported height and weight in future surveillance efforts.

Causal relationships can not be inferred from these cross-sectional data. Although data were based on self-reports, the measures in the student questionnaire have been previously demonstrated to be reliable and valid (16), and honest reporting was encouraged by ensuring confidentiality during data collection.

Because of the increasing prevalence of obesity among youth populations, a better understanding of sedentary behavior is necessary. We found that even when controlling for individual student characteristics, the characteristics of the school a student attends were associated with his or her likelihood of having high screen time. Future research should evaluate whether the optimal population impact for school-based activity promotion might be achieved most economically if interventions selectively target the schools that are putting students at the greatest risk for being sedentary.

## Figures and Tables

**Table 1:** Student-Level Characteristics (N = 2,449), Play-On Study, Ontario, Canada, 2007-2008

**Characteristic**	Boys, No. (%), n = 1,152^a^	Girls, No. (%), n = 1,277^a^	χ^2^, *df*	*P* Value
**Screen time per day**				
<1 hour per day	211 (19)	312 (25)	30.4, 2	<.001
1 to 3 hours per day	711 (63)	796 (63)		
>3 hours per day	214 (19)	148 (12)		
**PA level^b^**				
High active	220 (20)	171 (14)	15.1, 2	<.001
Moderately active	731 (65)	868 (69)		
Low active	175 (16)	214 (17)		
**Weight status^c^**				
Normal weight	448 (40)	460 (37)	44.3, 3	<.001
Overweight	116 (10)	70 (6)		
Obese	70 (6)	40 (3)		
Missing	480 (43)	669 (54)		
**Number of close friends who are physically active **				
0 to 2	109 (10)	127 (10)	0.1, 1	.76
3 or more	1,016 (90)	1,135 (90)		
**Parental encouragement of PA**				
Encourage	516 (45)	604 (48)	2.5, 2	.28
Strongly encourage	517 (45)	558 (44)		
Do not encourage	106 (9)	99 (8)		
**Parental support of PA**				
Supportive	394 (35)	402 (32)	2.8, 2	.24
Very supportive	707 (62)	835 (66)		
Unsupportive	31 (3)	33 (3)		
**Grade**				
5	268 (23)	326 (26)	42.0, 3	.24
6	306 (27)	331 (26)		
7	297 (26)	347 (27)		
8	281 (24)	273 (21)		
**Race/ethnicity**				
White	699 (62)	694 (55)	9.8, 1	.002
Nonwhite	437 (38)	563 (45)		

Abbreviations: df, degrees of freedom; PA, physical activity.

a Numbers may not add to total because of missing values. Percentages may not total 100 because of rounding.

b See Methods for definition of PA levels.

c See Methods for definition of weight status categories; body mass index values used to determine weight status have been adjusted for age and sex.

**Table 2:** School-Level Characteristics (N = 30), Play-On Study, Ontario, Canada, 2007-2008

Characteristic	Phase of Implementation^a^		

	Initiation, n	Action, n	Maintenance, n
**Healthy physical environment**			
Student access to a variety of facilities on or off school grounds during school hours	1	12	17
Availability of physical activities during inclement weather	16	12	2
Student access to facilities and equipment outside of school hours	10	19	1
Support for active transportation to and from school	7	14	9
Overall score for this indicator^b^	10	20	0
**Instruction and programs**			
Implementation of daily PA	0	24	6
Time spent per week engaged in PA during physical education classes	28	1	1
Classes taught by a qualified physical education specialist	26	4	0
Availability and use of intramural or club activities	24	4	2
Consistency of intramural programming across grade divisions and seasons	11	13	6
Availability and use of interschool programs	16	13	1
Consistency of interschool programming across seasons	5	0	25
Overall score for this indicator^b^	22	8	0
**Supportive social environment**			
Emphasis placed on maximizing participation in PA through school programs	3	7	20
Incorporation of PA into other school subjects	6	19	5
Special recognition of students who participate in school physical activities	3	6	21
Formal collection of suggestions from the school community about PA at school	18	9	3
Promotion of PA programs and events for students, families, and school staff	7	9	14
Use of PA as a reward, not as discipline	12	12	6
Presence of written policies or practices that support PA	6	16	8
Overall score for this indicator^b^	10	20	0
**Community partnerships**			
Support available for school staff involved with PA	0	9	21
Connection to community resources	6	4	20
Overall score for this indicator^b^	5	8	17

Abbreviation: PA, physical activity.

a Phases of implementation are based on Foundations for a Healthy School (18). Initiation is defined as falling short of meeting the recommendations related to school capacity for PA; action is defined as meeting the recommendations in several but not all areas; maintenance is defined as consistently meeting or exceeding the recommendations.

b Represents an overall score calculated for each school based on the combined responses to its indicator scores for each Foundations for a Healthy School component.

**Table 3:** Student- and School-Level Factors Associated With Screen Time, Play-On Study, Ontario, Canada, 2007-2008

Characteristic	Model 1^a^, Moderate Screen Time vs Low Screen Time		Model 2^b^, High Screen Time vs Low Screen Time	

	OR^c^ (95% CI)	*P* Value^d^	OR^c^ (95% CI)	*P* Value^d^
**Student level**				
**PA level^e^**				
Low active	1 [Reference]			
Moderately active	1.27 (0.94-1.72)	.14	1.12 (0.73-1.72)	.61
High active	1.36 (0.92-2.01)	.15	1.51 (0.86-2.64)	.17
**Weight status^f^**				
Normal weight	1 [Reference]			
Overweight	0.94 (0.66-1.34)	.74	1.22 (0.74-2.00)	.45
Missing	1.03 (0.81-1.30)	.81	1.04 (0.74-1.71)	.81
**Number of close friends who are physically active**				
0 to 2	1 [Reference]			
3 or more	1.42 (1.03-1.96)	.04	1.04 (0.63-1.71)	.88
**Parental encouragement of PA**				
Encourage	1 [Reference]			
Strongly encourage	0.62 (0.49-0.78)	<.001	0.66 (0.38-1.13)	.15
Do not encourage	1.47 (0.89-2.44)	.15	1.96 (1.07-3.61)	.047
**Parental support of PA**				
Supportive	1 [Reference]			
Very supportive	0.84 (0.65-1.08)	.12	0.60 (0.42-0.85)	.009
Unsupportive	0.74 (0.33-1.66)	.48	1.91 (0.77-4.73)	.18
**Sex**				
Girls	1 [Reference]			
Boys	1.39 (1.12-1.73)	.009	2.21 (1.61-3.02)	<.001
**Race/ethnicity**				
Nonwhite	1 [Reference]			
White	1.06 (0.85-1.33)	.61	0.70 (0.50-0.98)	.046
**School level**				
**Emphasis placed on maximizing participation in PA through school programs^g^**				
Initiation	NC	NC	1 [Reference]	
Action	NC		0.46 (0.23-0.95)	.04
Maintenance	NC		0.48 (0.19-1.24)	.15

Abbreviations: OR, odds ratio; CI, confidence interval; PA, physical activity; NC, not calculated.

a Model 1: 1 = moderate screen time (n = 1,448), 0 = low screen time (n = 498).

b Model 2: 1 = high screen time (n = 345), 0 = low screen time (n = 498).

c Odds ratios adjusted for all other variables in the table and controlled for grade.

d Calculated by using *t* test.

e See Methods for definition of PA levels.

f See Methods for definition of weight status categories.

g Phases of implementation are based on Foundations for a Healthy School (18). Initiation is defined as falling short of meeting the recommendations related to school capacity for PA; action is defined as meeting the recommendations in several but not all areas; maintenance is defined as consistently meeting or exceeding the recommendations. Because there was no significant between-school variability in moderate screen time identified, no school characteristics were included in Model 1.
